# Clinical efficacy of high-flow nasal humidified oxygen therapy in patients with hypoxemia

**DOI:** 10.1371/journal.pone.0216957

**Published:** 2019-06-06

**Authors:** Qiliang Hou, Zhigang Zhang, Ting Lei, Maozhou Gan, Xiangjun Wu, Weigang Yue, Bin Li, Lin Deng, Hongchang Gong

**Affiliations:** 1 Department of Critical Care Medicine, Guanghan People's Hospital, Sichuan, China; 2 Department of Critical Care Medicine, The First Hospital of Lanzhou University, Lanzhou, China; 3 Department of Obstetrics & Gynecology, The First Hospital of Lanzhou University, Lanzhou, China; 4 Guanghan People's Hospital, Sichuan, China; Cleveland Clinic, UNITED STATES

## Abstract

To evaluate the effectiveness of high-flow nasal humidified oxygen (HFNHO) therapy in patients with mild hypoxemia after extubation. This study included 316 patients with mild hypoxemia after extubation from May 2016 to May 2018 from two intensive care units in China. Compare the effects of the Venturi Mask and High-Flow Nasal Humidified Oxygen (HFNHO) therapy on Heart Rate (HR), Respiratory Rate (RR), Oxygen Saturation (SpO_2_), Oxygen Partial Pressure (PO_2_), Partial Pressure Of Carbon Dioxide (PCO_2_), Oxygenation Index (PO_2_/FiO_2_) after extubation, the use of noninvasive mechanical ventilation and tracheal intubation after treatment failure were observed and recorded. Patients have both lower HR and RR than those who received mask treatment (75.4±18.5 vs. 83.0±20.4, p = 0.0004; 18±6.5 vs. 23.6±10.3, p<0.001, respectively). There was significant difference between those who had HFNHO and mask administration’s SpO_2_ and PO_2_ (94.1±6.4 vs. 87.5±1.5, p<0.001; 88.16±2.9 vs. 77.3±2.3, p<0.001, respectively). For the HFNHO group, patients had lower PCO_2_ with the mask group. (41.3±0.99 vs 42.2±1.2, p<0.001). On the other hand, the levels of PO_2_/FiO_2_ was significantly higher in the HFNHO Group, (181.0±8.3 vs. 157.2±4.9, p<0.05). We concluded HFNHO therapy could significantly relieve the symptoms of dyspnea, improve oxygenation, reduce the use of noninvasive mechanical ventilation and reduce the rate of secondary tracheal intubation in patients with mild hypoxemia after extubation.

## Introduction

The overall goal of oxygen therapy administration is to maintain adequate tissue oxygen supply, high-flow nasal humidified oxygen (HFNHO) therapy refers to a novel noninvasive ventilation and oxygen therapy method that uses a high flow of air containing a certain concentration of oxygen; compare to the regular invasive ventilation, HFNHO shows its superiority in the direct air supplied to patients via nasal cannulas that are not necessarily airtight[1–4]. HFNHO has the ability to output warm air containing a constant oxygen concentration of 21–100% at a temperature of approximately 37°C and a relative humidity of 100%[5–7], ensure normal airway mucociliary function and promote the elimination of sputum[8]. HFNHO therapy is superior to conventional methods using ordinary nasal cannulas or a regular mask as well [6, 7, 9]. The maximum output flow rate can reach 50 L/min, during inhaling, HFNHO reduces the power consumption of respiration and generates a certain positive pressure[10], which similar to positive end-expiratory pressure, could elevate the functional residual capacity as well[7, 11]. HFNHO therapy is an alternative to noninvasive positive pressure ventilation in adults with mild hypoxemia[12]. Previous HFNHO investigations have dominantly done within respiratory failure patients[13–17]; however, to date, the Effect of HFNHO in patients with mild hypoxemia was rarely reported. This study aimed to investigate the correlation between HFNHO therapy and the frequency of noninvasive ventilation and the proportion of patients who received secondary tracheal intubation.

## Methods

This was a prospective cohort study. The outcome was whether patients were able to receive HFNHO therapy with mild hypoxemia after tracheal extubation. The study protocol was approved by the Ethics Committee of The First Hospital of Lanzhou University of China, and informed verbal consent was obtained from all participants or immediate family member or legally-authorized representative with witness. The authors had access to information that could identify individual participants during or after data collection.

The subjects consisted of 316 patients with mild hypoxemia after tracheal extubation who were admitted to two intensive care units from May 2016 to May 2018. The patients were randomly divided into two groups, 156 patients in the control group and 160 patients in the experimental group. In the control group, patients received oxygen supplementation via an ordinary mask after tracheal extubation. And the patients received HFNHO therapy after tracheal extubation in the experimental group; the total treatment period was 120±14.9h. The patients who fulfilled the following criteria were included: (1) at least one dyspnea-related symptom such as shortness of breath, three-concave sign, cyanosis, wheezing and laryngeal stridor after mechanical ventilation withdrawal and (2) PO_2_ <80 mmHg by the arterial blood gas analysis after mechanical ventilation withdrawal (met the diagnostic criteria for hypoxemia). The main exclusion criteria were: (1) unstable cyclic indicators such as heart rate (HR) and blood pressure and (2) low consciousness that affected autonomous respiration.

All patients underwent physical examination, including anthropometric measures (heights/weights), body mass index (BMI) was calculated, laboratory testing, level of serum sodium (Na), potassium (K), hemoglobin, C-reactive protein (CRP), brain natriuretic peptide (BNP), echocardiography, and there were no significant differences in the age and gender distribution between the examined subgroups (Table 1).

**Table 1:** Summary statistics for demographics.

Indicator (n = 316)	HFNHO group (n = 160)	Mask group (n = 156)	*p*-value
General information			
Male/Female (number of cases)	83/77	84/72	0.397
Age (years)	48.8±14	50.0±13.7	0.262
BMI (kg/m^2^)	20.8±1.2	21.1±0.9	0.096
BP (Systolic/Diastolic)(mmHg)	116.7±16.4 /73.8±11.5	119±13.3/78.8±15	0.14/0.01
Treatment Period (h)	120±14.9	125±16	0.3636
Laboratory Data			
Hb (g/dL)	124.8±13.4	122.1±15.7	0.07
Sodium (mEq/L)	140.8±9.6	139.6±10.5	0.35
Potassium (mmol/L)	4.1±0.7	4.1±0.6	0.88
CRP	6.2±1.3	6.2±1.3	0.84
BNP (pg/mL)	89±20	92±18	0.457
Diseases			
Severe Pneumonia	67	69	>0.05
Severe Acute Pancreatitis	24	22	>0.05
Esophageal Carcinoma (underwent radical surgery)	16	18	>0.05
Heart Failure	13	15	>0.05
LVEF, n (%)			
LVEF <40	9(69%)	11(73%)	>0.05
40<LVEF <30	4(31%)	4(27%)	>0.05
Sleep Apnea Syndrome	9	8	>0.05
Multiple Injuries	25	24	>0.05
Other	6	0	
Additional Therapy/ Medications			
Antibiotic Medications	145	139	>0.05
Blood Transfusion	7	6	>0.05
Pain management	40	37	>0.05

BMI, body mass index; BP, blood pressure; LVEF, left ventricular ejection fraction; Hb, hemoglobin; CRP, C-reactive protein; BNP, brain natriuretic peptide. Statistics presented as Mean ± SD or N (column %).

Oxygen was supplied to the control group via an ordinary mask, and the inhaled flow rate was adjusted according to the oxygenation condition of each patient. The new high-end ventilator (Drager-C300/C500, Germany) in an intensive care unit delivered the HFNHO therapy to patients in the experimental group. The ventilator can adjust the oxygen concentration (FiO_2_) from 21% to 100%, flow rate set from 10 to 50 L/min, and the temperature range set from 32 to 41°C. The MR850 humidifier, oxygen delivery tubes, and high-flow nasal cannulas were purchased from Fisher & Paykel (New Zealand). Nasal cannulas were secured onto the head of each patient using the attached head strap during oxygen inhalation. The parameters were adjusted according to the respiratory condition of each patient. The Heart Rate (HR), Respiratory Rate (RR), Oxygen Saturation (SpO_2_), Oxygen Partial Pressure (PO_2_), Partial Pressure of Carbon Dioxide (PCO_2_), Oxygen Index (PO_2_/ FiO_2_) after oxygen therapy, the application of noninvasive mechanical ventilation and tracheal intubation after treatment failure were observed and recorded for patients in the two groups.

Data processing was performed using the GraphPad Prism 8.0 software (San Diego, CA, USA). Between-group differences in quantitative variables were assessed with the t-test (for a normal distribution) or the Mann-Whitney test (for skewed variables). The chi-square test was used as appropriate for the analysis of between-group differences in categorical variables. Univariate linear regression was used to assess the association between inter-quantitative variables. The significance level was set at a *p* value < 0.05.

## Results

**Fig 1:**
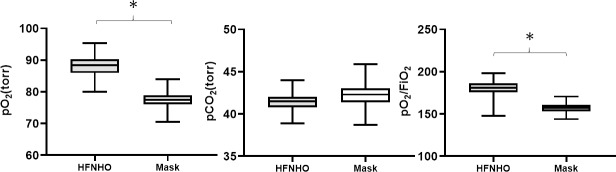
The difference between HFNHO and mask administration group’s SpO_2_ (Fig 1A), PO_2_ (Fig 1B), and PO_2_ /FiO_2_ (Fig 1C). Data are mean value ± SD; *p < 0.05.

Data from 316 consecutive patients admitted between May 2016 to May 2018, were included in this study. There were 167 (83 vs. 84) males and 149 (77 vs. 72) females, age ranging in 49±13.8 years; average BMI was 20.8±1.2 vs. 21.1±0.9 kg/m^2^, blood pressure was ranking from 116.7±16.4/73.8±11.5 mmHg vs 119±13.3/78.8±15 mmHg. No significant differences were noted in the Hb, Na, K, CRP, and BNP, between the two groups (p >0.05), and the treatment period of two groups didn’t show the significant difference (Table 1). During the hospitalization and oxygen administration: in the HFNHO group, patients have both lower HR and RR than those who received mask treatment (75.4±18.5 vs. 83±20.4, p = 0.0004; 18±6.5 vs. 23.6±10.3, p = 0.0004, respectively) (Table 2). There was significant difference between those who had HFNHO and those who had mask administration’s SpO_2_ and PO_2_ (94.1±6.4 vs. 87.5±1.5, p<0.001; 88.16±2.9 vs. 77.3±2.3, p<0.001, respectively) (Fig 1A). For the HFNHO group, patients who had lower PCO_2_ with the mask group. (41.3±0.99 vs 42.2±1.2, p<0.001) (Fig 1B). On the other hand, the levels of PO_2_ / FiO_2_ was significantly higher in the HFNHO Group, (181.0±8.3 vs. 157.2±4.9, p<0.001) (Fig 1C).

**Table 2:** Summary Statistics by Groups (HFNHO vs MASK).

	Overall (N = 316)		HFNHO (N = 160)		MASK (N = 156)		
Factor	N	Statistics	n	Statistics	n	Statistics	*p*-value
HR(/min)	316	79.1±19.8	160	75.4±18.5	156	83±20.4	***0*.*0004***^***a***^
RR(/min)	316	20.8±9.0	160	18±6.5	156	23.6±10.3	***<0*.*001***^***a***^
SpO_2_(%)	316	90.8±5.6	160	94.1±6.4	156	87.5±1.5	***<0*.*001***^***a***^
PO_2_	316	82.74±6.08	160	88.16±2.9	156	77.3±2.3	***<0*.*001***^***a***^
PCO_2_	316	41.8±1.2	160	41.3±0.99	156	42.2±1.2	***<0*.*001***^***a***^
PO_2_/FiO_2_	316	168.9±13.5	160	181.0±8.3	156	157.2±4.9	***<0*.*05***^***a***^

HR, Heart Rate; RR, Respiratory rate; SpO_2_, oxygen saturation; PO_2_, oxygen partial pressure; pCO_2_, the partial pressure of carbon dioxide; PO_2_/FiO_2_/, oxygenation index. Statistics presented as Mean ± SD. p-values: a = two-sample t-test.

The frequency of noninvasive ventilation and the proportion of patients who received secondary tracheal intubation in the investigated groups are listed in Table 2. while noninvasive ventilation and secondary tracheal intubation were significantly increased in the subgroups of patients with mask compared to HFNHO group (p <0.05 in both groups) (Table 3). Furthermore, the 60 days Mortality was not significantly decreased in the subgroups of patients with HFNHO compared to the subgroups of patients with mask administration (*p* >0.05).

**Table 3:** HFNHO and MASK group Prognosis and Mortality.

	Noninvasive Ventilation (%)	Secondary Tracheal Intubation (%)	Mortality (60day)
HFNHO	8(5)	6 (3.75)	9 (5.63)
Mask Group	19(12.2)	15 (9.62)	11 (7.05)
*p*	0.0225^b^	0.0364^b^	0.602^b^

Statistics presented p-values: b = chi-square test.

## Discussion

Hypoxemia is one of the key air exchange abnormalities associated with respiratory disease. It denotes a blood oxygen concentration or partial pressure of oxygen (PO2) below normal[18, 19]. Both pulmonary and extrapulmonary disorders cause hypoxemia [20, 21]. Examples include pneumonia, chronic obstructive pulmonary disease (COPD), acute respiratory distress syndrome/acute lung injury (ARDS/ALI) and congestive heart failure (CHF). Untreated hypoxemia jeopardizes the heart and brain. Thus, it is imperative to diagnose and treat hypoxemia in the first place.

In the patients suffering the hypoxemic, oxygen therapy is serving as the first-line treatment[22]. Different devices can be used to deliver oxygen therapy, such as nasal cannulas, nonrebreathing masks, and bag-valve masks. But the fraction of inspired oxygen obtained by using these methods varied a lot depending on the patients’ breathing pattern and other limiting factors. In addition, treatment with these devices could result in several results that make patients feel uncomfortable, such as dry mouth and dry nose etc. these side effects of oxygen therapy could be relieved dramatically by heating and humidifying the oxygen-air mixture. Over the past two decades, devices that deliver heated and humidified oxygen-air at high flow rates through a nasal cannula were developed as an alternative to low/medium flow devices. The high flow rate of the oxygen-air mixture and the adjustable oxygen concentration in the mixture improve the FiO_2_ in patients. High flow rate nasal humidified oxygen therapy becomes more and more used to treat hypoxemia in intensive care units and other medical environments.

Although the application of HFNHO therapy in adult patients is relatively new, the therapy has developed rapidly as a new and effective oxygen therapy method [23, 24]. Compared with traditional nasal cannulas and masks, HFNHO therapy better improves oxygenation, increases oxygen partial pressure, reduces anatomical dead space in adult patients [25], ensures adequate airway humidification, improves ventilation and relieves hypoxemia in patients[26]. Although there is still a certain gap between this oxygen supply method and noninvasive positive pressure ventilation[26], the early application of HFNHO therapy in hypoxemia patients can reduce the occurrence of respiratory failure[27, 28] and avoid the use of noninvasive ventilation and the occurrence of secondary tracheal intubation[29–31]. In addition, HFNHO therapy has a clear advantage in comfort and compliance compared with noninvasive ventilation[27]. The operation of HFNHO therapy is more straightforward and thus makes nursing more comfortable. Under the premise of satisfying the ventilatory oxygenation requirement[32], HFNHO therapy is a better choice than conventional methods.

In HFNHO therapy, the required oxygen concentration, flow rate, and temperature can be adjusted according to the respiratory condition of each patient, thus providing individualized treatment[7, 33, 34]. In this study, after HFNHO therapy was conducted in patients with mild hypoxemia after tracheal intubation, the HR and RR of patients in the HFNHO group decreased with time and returned to normal values, and they were significantly lower than those of the control group (P<0.05). HFNHO therapy significantly relieved respiratory distress syndrome and improved oxygenation. In addition, based on blood gas analysis indicators, HFNHO therapy significantly increased the oxygen partial pressure in the blood and the oxygenation index (P<0.05) to ensure oxygen supply to various organs and tissues and delay the progression of respiratory failure. Moreover, the high flow provided by HFNHO therapy can overcome airway resistance when the patient inhales [35], reduce the power consumption of respiratory muscles and relieve respiratory muscle fatigue. Meanwhile, the high-flow air creates resistance when the patient exhales[36], which causes positive pressure to form in the patient's airway[11]. The positive pressure, which is similar to positive end-expiratory pressure, can increase the amount of functional residual capacity, promote alveolar recruitment and improve oxygenation[37].

Previous studies about HFNHO were dominantly done within acute respiratory failure patients. Effect of HFNHO in patients with mild hypoxemia was rarely reported. In our study, it was found that HFNHO improved the outcomes of patients with mild hypoxemia than the oxygen therapy with an ordinary mask, which was consistent with the results of HFNHO in acute respiratory failure patients. Our results provided evidence of a better treatment result from HFNHO than common oxygen therapy, indicating that HFNHO might be a better option to treat mild hypoxemia than commonly used, mask-based oxygen therapy.

Although HFNHO has advantages over conventional mask-based oxygen therapy, several aspects that are still needed to be pointed out and further discussed include: First, HFNHO does not require patient cooperation, it is generally better tolerated and easier to use and requires less equipment and lower nursing workload. On the other hand, just because its easy use and being well tolerated by patients, closer surveillance by caregivers becomes more critical and has to be enforced to ensure timely intubation if HFNHO fails. Second, it needs to be well considered whether the HFNHO is the best option for a specific patient. For example, the application of HFNHO relieves the burden of respiratory muscles. But for some patients, exercising of their respiratory muscles is one of the requirements for their recovery. Thus, choosing the appropriate method to facilitate patients’ breath has to take a patient’s specific needs under consideration[20].

This study further shows that HFNHO therapy significantly reduced the frequency of noninvasive ventilation and the proportion of patients who received secondary tracheal intubation (P<0.05). This result indicates that the timely use of HFNHO therapy in hypoxemia patients avoids the use of noninvasive ventilation and reduces the occurrence of secondary tracheal intubation. At the same time, HFNHO therapy can ensure the thorough heating and humidification of the inhaled gas and thus normal airway mucociliary function to promote the elimination of sputum in the airway[38], such that the aggravation of dyspnea symptoms and respiratory failure due to viscous sputum-caused airway blockage can be avoided[4].

## Conclusion

HFNHO therapy is a developing type of noninvasive oxygen therapy for adults and can be used when normal nasal cannulas or masks fail to alleviate hypoxemia. HFNHO therapy can significantly relieve the symptoms of dyspnea, improve oxygenation, reduce the use of noninvasive mechanical ventilation and reduce the rate of secondary tracheal intubation in patients with mild hypoxemia after extubation.

## Supporting information

S1 Table STROBE statement—Checklist of items that should be included in reports of observational studies. (DOCX)

S1 File Original data in GraphpadPrism format. (PZFX)
